# Tunable Beam Steering Metasurface Based on a PMN-PT Crystal with a High Electro-Optic Coefficient

**DOI:** 10.3390/s25010055

**Published:** 2024-12-25

**Authors:** Huan Chen, Zixin Wang, Xin Chen, Junli Wang

**Affiliations:** 1 School of Physics, Xidian University, Xi’an 710071, China; 15059711556@139.com (Z.W.); xin.chen@xidian.edu.cn (X.C.); 2 Guangzhou Institute of Technology, Xidian University, Guangzhou 510555, China

**Keywords:** tunable metasurface, PMN-PT crystal, electro-optic effect, beam deflection

## Abstract

Existing tunable optical metasurfaces based on the electro-optic effect are either complex in structure or have a limited phase modulation range. In this paper, a simple rectangular metasurface structure based on a Pb(Mg_1/3_Nb_2/3_)O_3_-PbTiO_3_ (PMN-PT) crystal with high electro-optic coefficient of 120 pm/V was designed to demonstrate its electrically tunable performance in the optical communication band through simulations. By optimizing the structure parameters, a tunable metasurface was generated that can induce a complete 2π phase shift for beam deflection while maintaining relatively uniform transmittance. Simulations further demonstrated the electrical tunability of the beam deflection direction and operating wavelength of the metasurface. This tunable optical metasurface, with its simple and easily fabricated structure, can promote the development and application of multifunctional and controllable metasurfaces. Its adjustable beam deflection direction and operating wavelength may find applications in fields such as optical communication systems and imaging.

## 1. Introduction

Optical metasurfaces are ultrathin planar optical devices comprising artificially designed micro- and nano-structures [1,2]. Initially, once the optical metasurfaces were fabricated, their material properties and geometrical configurations remained immutable under external conditions [1,3,4,5,6,7,8,9,10]. Consequently, early metasurfaces lacked flexibility in regulating light waves, limiting their progression and utility. Presently, the demand for micro- and nano-photonic devices is growing in diverse applications, such as augmented reality/virtual reality (AR/VR), lidar, and autonomous driving. Tunable metasurfaces [11] offer a versatile platform for manipulating key aspects of light waves, including polarization, amplitude, and phase, which are pivotal in the mentioned applications.

In recent years, significant efforts have been directed toward developing tunable optical metasurfaces with multifunctionality through various control methods, including pressure, heat, electric current, and external illumination. These approaches enable the dynamic modulation of light waves [11]. Notably, rapid advancements in micro- and nano-fabrication techniques, including laser processing, lithography, photo-curing molding, 3D printing, and laser stripping, have provided conditions for manufacturing at micro- and even nano-scales. Driven by industrial demands and technological support, tunable metasurfaces are evolving toward higher control speeds, increased depth in phase and amplitude modulation, enhanced efficiency, and reduced power consumption [11]. The principles of current tunable optical metasurfaces primarily fall into two categories: those relying on active materials and those achieved through mechanical actuation. Active materials, such as graphene [12,13,14], transparent conductive oxides [15,16,17,18], transition metal sulfides [19,20,21], phase-change materials [22,23,24,25,26,27,28,29,30], liquid crystals [31,32,33,34,35], and electro-optic crystals [36,37,38,39,40,41,42], have optical properties that can be modulated through applying external conditions such as heat, electricity, and light. Conversely, mechanical actuation alters the optical response by reconfiguring the geometric shape or spatial arrangement of the micro- and nano-structure units constituting the metasurface, often involving adjustments to mechanical structures or elastic deformation of flexible, stretchable materials [43,44,45].

Among these approaches, tunable metasurfaces employing phase-change materials exhibit versatile control methods, including optical, thermal, and electrical modulation. However, there is room for improvement in the control speed of phase-change material metasurfaces [22,23,24,25,26,27,28,29,30]. Tunable metasurfaces based on liquid crystals [31,32,33,34,35] encounter two primary challenges. Spatially, precise control necessitates pixel-by-pixel biasing of liquid crystals, requiring limited spacing between each electrode at the microscale, thereby constraining spatial integration. And temporally, liquid crystals exhibit switching times in the millisecond range, rendering their response relatively sluggish. Most graphene-based structures in tunable metasurfaces are complex, impeding practical applications [12,13,14]. Compared with those based on phase-change materials and liquid crystals, existing tunable metasurfaces based on the electro-optic effect have the advantages of fast adjustment speed and easy integration, but there are still some problems, such as complex structure and limited phase modulation range [36,37,38,39,40,41,42]. In order to solve these problems for existing tunable metasurfaces based on the electro-optic effect, in this paper, a straightforward rectangular metasurface structure was designed by employing a PMN-PT electro-optic crystal with a high refractive index and a high electro-optic coefficient of 120 pm/V [46,47]. Its electrically tunable performance in the optical communication band was proved by simulation. Utilizing this tunable metasurface structure, a beam-steering metasurface capable of achieving a complete 2π phase shift was designed through optimizing structure parameters and applying external voltage. The simulation results demonstrated that the beam deflection direction and operating wavelength of the metasurface were electrically adjustable. This uncomplicated and easily fabricated tunable metasurface holds the potential to advance the development and utilization of multifunctional, controllable metasurfaces, while also playing a significant role in reducing the cost of optical metasurface production and broadening the application scope of such metasurfaces.

## 2. Results and Discussion

### 2.1. Beam-Deflecting Metasurface Based on a PMN-PT Electro-Optic Crystal

**Figure 1:**
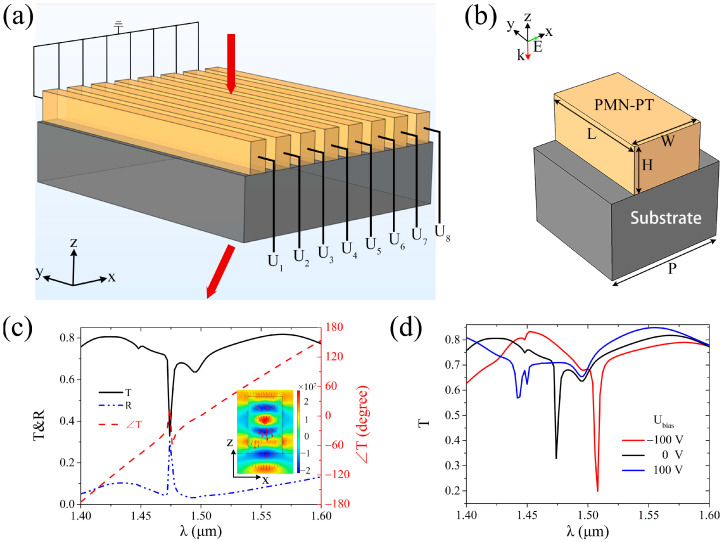
Simulation of the PMN-PT metasurface structure unit. (**a**) Schematic of the tunable beam-deflecting PMN-PT metasurface. The red arrows, from top to bottom, represent incident and refrected light, respectively. (**b**) Three-dimensional view of the structure unit. The dimensions include a length L = 1.5 μm, width W = 0.9 μm, and height H. The period P = L = 1.5 μm. During simulation, *x*-polarized light (green arrow) is incident on the metasurface from the negative *z*-direction (red arrow). (**c**) Transmission (black solid line and red dashed line) and reflection (blue dot-dashed line) spectra of the metasurface in optical communication band. The inset shows the distributions of the electric field *E_x_* and energy flow (red arrows) of the metasurface structure at the incident of 1.5 μm *x*-polarized light. The color legend specifies the electric field *E_x_* (unit: V/m). (**d**) Spectra of the resonance shifting with the applied voltages −100 V (red), 0 V (black), and +100 V (blue).

According to the literatures [46,47], high-refractive index PMN-PT single crystals exhibit noteworthy characteristics, including high transmittance and a high electro-optic coefficient in the optical band. Consequently, they can be used to achieve electrically adjustable beam deflection manipulation, as shown in Figure 1a. Firstly, this study harnesses a PMN-PT electro-optic crystal to craft an electrically tunable metasurface structure in the optical communication band, as illustrated in Figure 1b. The metasurface structure consists of an upper layer comprising rectangular PMN-PT crystal blocks and a glass substrate. The dimensions of the PMN-PT crystal block include a length L = 1.5 μm, a width W = 0.9 μm, and a height H, with a structural unit period P = L = 1.5 μm. The refractive index of the PMN-PT crystal is defined as *n* = *n*_0_ − 0.5*n*_0_^3^*γ*_13_*E_y_*, where *n*_0_ = 2.6 and *γ*_13_ = 120 pm/V, and *E_y_* signifies the electric field generated along the *y*-direction in the PMN-PT crystal block due to the applied external voltage U_bias_ (specifically, the face of the block in the positive *y*-direction is grounded, and the potential of the opposite face in the negative *y*-direction is set to U_bias_). The relative permittivity of the glass substrate is fixed at 3.75. As described in the literature [46], to measure the electro-optic coefficient *γ*_13_ of the PMN-PT crystal, the applied electric field is along the [001] direction, and the light beam is along the [110] direction. Therefore, in order to utilize the electro-optic coefficient *γ*_13_, the [001] direction of the PMN-PT crystal used in this paper is along the *y*-direction, while its [110] direction is along the *z*-direction. The boundaries of the structure unit along the *x*- and *y*-directions are governed by Floquet periodic conditions, while the *z*-direction is equipped with incident and exit ports, incorporating perfect matched layers at the ports. Since the length L of the structure is equal to its period P, and the boundary conditions in the *y* direction are periodic boundaries, the length L has no effect on the optical response of the structure when no external voltage is applied. However, when external voltages are applied, to achieve the same performance, once the length L changes, the external voltages also change in equal proportion. Consequently, the beam-deflecting ability of the metasurface composed of this structure is realized only in the *xz* plane.

The transmission and reflection spectra of the metasurface are simulated via COMSOL Multiphysics 5.5 software, with U_bias_ = 0 V and H = 1.5 μm. The *x*-polarized light is perpendicularly incident onto the metasurface from the negative *z*-direction, and the resultant transmission and reflection spectra of the metasurface are depicted in Figure 1c. The transmission spectrum reveals a transmission dip near 1.5 μm, signifying resonance in the optical communication band. Considering the reflectance spectrum curve in this figure, the transmission (T) dip is caused by strong reflection (R) and absorption (defined as A = 1 − T − R). Due to the dimensions of the metasurface structure being approximately 1.5 μm, this enables the metasurface to experience Mie resonance in the optical communication band and further causes the metasurface to generate a strong reflection around 1.5 μm. The inset in the figure shows the distributions of the electric field *E_x_* and energy flow of the metasurface structure at the incident of 1.5 μm *x*-polarized light. The color legend indicates the electric field *E_x_*, and the red arrows indicate the energy flow. Both the electric field distribution and energy flow corroborate the presence of localized resonance within the metasurface structure, thus substantiating the capacity of the rectangular PMN-PT metasurface structure for controlling the phase of light waves. Figure 1d shows spectral shifts of the resonance for U_bias_ = ±100 V. As seen, the resonance, namely the lowest transmission dip, red shifts with negative applied bias and blue shifts with positive applied bias, demonstrating that the rectangular PMN-PT metasurface structure is electrically tunable in the optical communication band.

**Figure 2:**
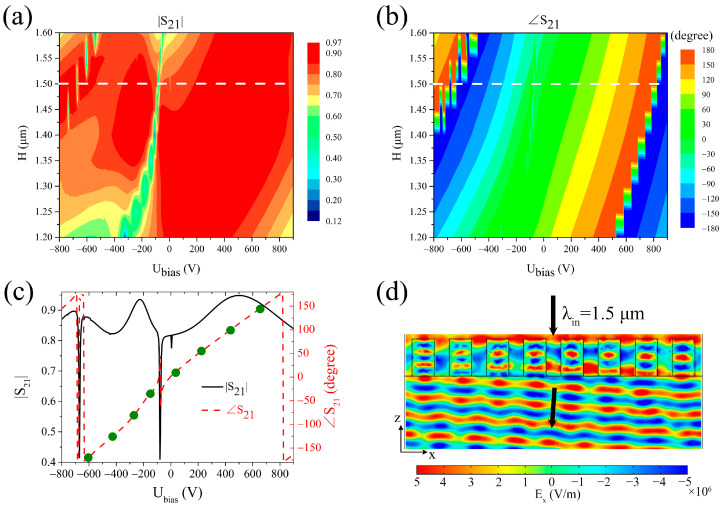
Simulation of the beam-deflecting metasurface. The influences of rectangular block height H and external voltage U_bias_ on the transmission coefficient S_21_ (**a**) amplitude and (**b**) phase are depicted. The position H = 1.5 μm is indicated by the white dashed line. (**c**) Relationships between the transmission coefficient S_21_ amplitude (black solid line) and phase (red dashed line) and the external voltage U_bias_ corresponding to the white dashed lines in (**a**,**b**). The green dots indicate the eight data points where the phase difference between adjacent points is 45°. (**d**) Electric field *E_x_* (specified by the bottom color legend) distribution of the transmitted light when the *x*-polarized light with λ = 1.5 μm is normally incident onto the metasurface. The black arrows, from top to bottom, indicate the directions of the incident and transmitted light waves, respectively.

Using the aforementioned rectangular PMN-PT metasurface structure, this study aimed to achieve complete 2π phase tunability while maintaining a relatively small external voltage value U_bias_ and uniform transmittance by optimizing the structure parameters and adjusting the external voltage. Since the metasurface structure is already operating in the optical communication band, no adjustments were made to its lateral structure parameters. The aim was exclusively achieved by optimizing the height H of the PMN-PT rectangular blocks. When *x*-polarized light was incident perpendicularly from the negative *z*-direction at wavelength λ = 1.5 μm, the relationship between the transmission coefficient S_21_ (the square of its modulus represents the transmittance) and the external voltage U_bias_ was established through varying the height H, as depicted in Figure 2a,b. Through analyzing these data, a height H = 1.5 μm (indicated by the white dashed line in the figures) was selected as the optimal value. At this specific height, complete 2π phase tunability could be achieved with a relatively small external voltage, and the amplitude fluctuation range of the transmission coefficient remained small (0.82~0.95, with certain lower amplitude regions skipped through adjusting the external voltage), ensuring uniform transmittance. Figure 2c shows the curves corresponding to the white dashed line in Figure 2a,b, i.e., the relationship between the transmission coefficient and external voltage at H = 1.5 μm. In this figure, it is found that, when the external voltage was a certain value, the amplitude of the transmission coefficient decreased greatly. This indicates that, when the external voltage is adjusted to a certain value, the refractive index of the PMN-PT crystal is changed just enough to cause the metasurface structure to resonate at this wavelength (extremely narrow resonance band, as shown in Figure 1c), which greatly reduces the transmittance of the metasurface structure. The green dots in the figure represent eight data points on the transmission coefficient phase curve, where the phase difference between adjacent points was 45°. Consequently, selecting the eight external voltages corresponding to these green dots enabled complete 2π phase shift of the incident plane wave via the metasurface structure.

A metasurface comprising eight identical structure units with H = 1.5 μm was constructed, with sequential application of eight external voltages (−605 V, −425 V, −270 V, −150 V, 35 V, 225 V, 435 V, 655 V) to these units. The electric field *E_x_* distribution of the transmitted light was simulated when the *x*-polarized plane wave with wavelength λ = 1.5 μm was vertically incident on the metasurface along the negative *z*-direction, as shown in Figure 2d. The black arrows in the figure denote the directions of the incident and transmitted light waves. In accordance with the generalized Snell’s law [1],

$$\sin(\theta_{t})n_{t}-\sin(\theta_{i})n_{i}=\frac{\lambda }{2\pi }\frac{d\phi }{dx},$$

where $\theta_{i}$ and $\theta_{t}$ represent the angles of incidence and refraction, respectively, $n_{i}$ and $n_{t}$ represent the refractive indexes of incidence and transmission space materials, respectively, λ represents the wavelength of incident light, and $\frac{d\phi }{dx}$ represents the constant phase gradient, and the theoretical refraction angle is 3.70° (in this paper, the transmitted space material is set as the glass substrate material, so the refractive index of glass substrate is substituted into the generalized Snell’s law), which is consistent with the deflection angle of 3.66° measured from Figure 2d. Here, the deflection angle obtained from the simulation was directly measured by the transmitted electric field using a protractor, so its value was bound to be affected by measurement errors and simulation errors, inevitably resulting in a deviation between the deflection angle calculated by the generalized Snell’s law and that measured from the simulation result.

**Figure 3:**
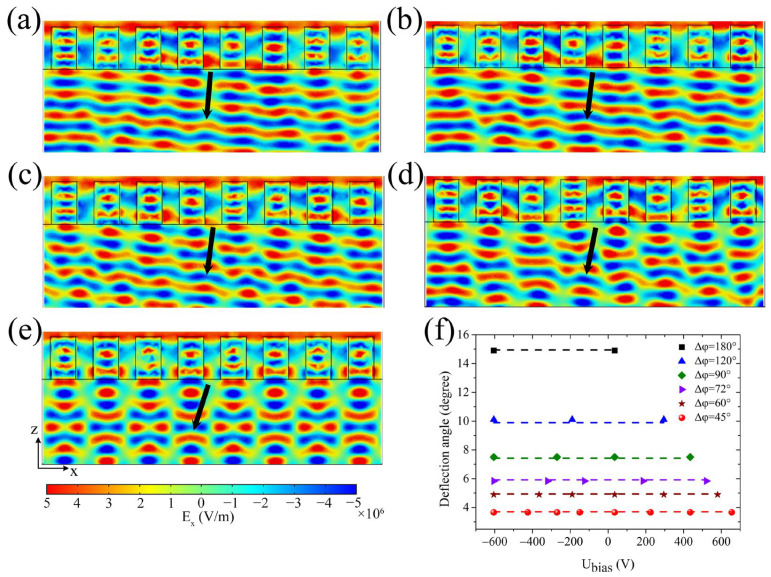
Regulation of the deflection direction for the beam-deflecting metasurface. (**a**–**e**) Electric field *E_x_* distribution of the transmitted light for *x*-polarized light at a wavelength of λ = 1.5 μm, incident perpendicularly onto the metasurface, when the phase difference between adjacent structure units is 60°, 72°, 90°, 120°, or 180°. (**f**) Relationship between the deflection angle and the U_bias_ voltages (actually the phase difference Δφ between adjacent units). The dashed line indicates the theoretical value of the deflection angle.

Furthermore, it becomes possible to manipulate the phase gradient of the metasurface by modulating the external voltage, thereby controlling the deflection direction of the transmitted light as desired. The data presented in Figure 2c indicate that other deflection angles can be obtained by adjusting the external voltages to alter the phase difference of the transmitted light between adjacent units. The metasurface is still composed of eight identical structure units (H = 1.5 μm). By adjusting the external voltages of the structure units, the phase difference between adjacent units of the metasurface was changed to 60°, 72°, 90°, 120°, and 180°. In other words, the external voltage U_bias_ is applied on the metasurface structure unit along the *y*-direction, and the external voltage of different structure units along the *x*-direction was different, resulting in a desired phase difference between adjacent structure units. The transmission electric field distributions of the metasurface with different phase differences were simulated and are shown in Figure 3a–e; the deflection angles were measured using a protractor, shown by the dots (the abscissas corresponding to these dots are the external voltages applied to the structure units in turn, and when the number of voltages is less than the number of units, the voltages are cyclically applied to the structure units in turn) with different shapes and colors in Figure 3f. Specifically, for the metasurface in Figure 3a, these voltages (−605 V, −365 V, −190 V, 35 V, 295 V, 580 V, −605 V, −365 V) were applied sequentially to its structure units; for the metasurface in Figure 3b, these voltages (−605 V, −320 V, −125 V, 185 V, 520 V, −605 V, −320 V, −125 V) were applied sequentially to its structure units; for the metasurface in Figure 3c, these voltages (−605 V, −270 V, 35 V, 435 V, −605 V, −270 V, 35 V, 435 V) were applied sequentially to its structure units; for the metasurface in Figure 3d, these voltages (−605 V, −190 V, 295 V, −605 V, −190 V, 295 V, −605 V, −190 V) were applied sequentially to its structure units; for the metasurface in Figure 3e, these voltages (−605 V, 35 V, −605 V, 35 V, −605 V, 35 V, −605 V, 35 V) were applied sequentially to its structure units. In these scenarios, the incident light was *x*-polarized at a wavelength of 1.5 μm. For comparison, Figure 3f contains the results when the phase difference between adjacent units was 45°. And this figure shows, the measured deflection angles of the metasurface with different phase differences were different, spanning 3.66°–14.90°, but were almost consistent with the theoretical values (corresponding to the dashed line) calculated by the generalized Snell’s law [1]. Therefore, increasing the phase difference between adjacent structure units by adjusting the external voltages increases the beam-deflecting angle of the metasurface. Thus, these simulations underscore the capability to arbitrarily control the deflection direction of light by simply adjusting the external voltage applied to the PMN-PT rectangular blocks, without necessitating any modifications to the structure or geometric parameters of the metasurface.

### 2.2. Tunable PMN-PT Beam-Deflecting Metasurfaces

**Figure 4:**
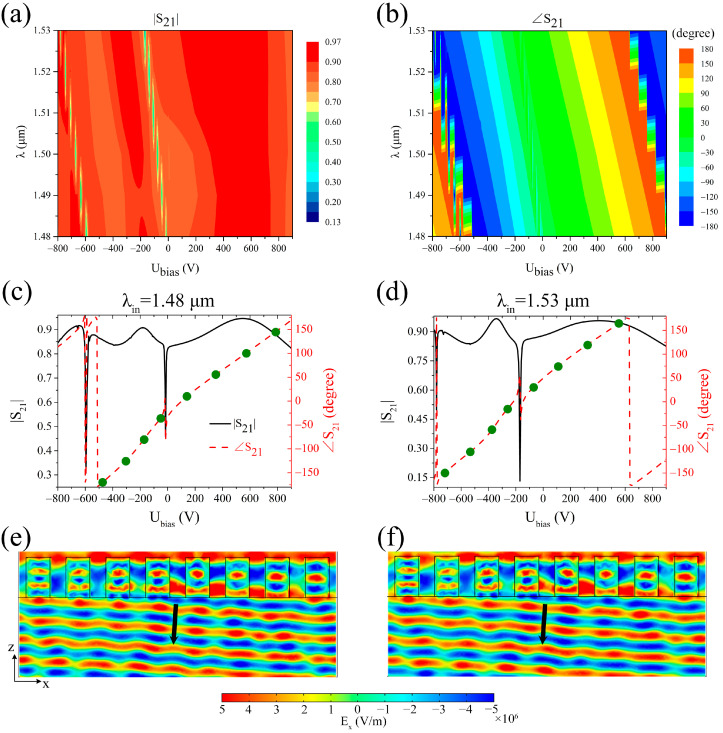
Tunable PMN-PT beam-deflecting metasurface. Relationships between the transmission coefficient S_21_ (**a**) amplitude and (**b**) phase with incident light wavelength λ and external voltage U_bias_ are displayed. (**c**,**d**) Relationships between the S_21_ amplitude (black solid line) and phase (red dashed line) with external voltage U_bias_ for incident light wavelengths of λ = 1.48 μm and 1.53 μm, respectively. The green dots indicate the data points where the phase difference between adjacent points is 45°. (**e**,**f**) Electric field *E_x_* distribution of the transmitted light for the metasurface at incident light wavelengths of λ = 1.48 μm and 1.53 μm, respectively.

Building upon the previously discussed PMN-PT metasurface, which is renowned for its ability to precisely control the direction of beam deflection, here we investigate the relationship between the transmission coefficient S_21_ amplitude and phase in conjunction with the external voltage U_bias_ by varying incident light wavelengths, as depicted in Figure 4a,b. This investigation was carried out for *x*-polarized light, which was incident perpendicularly from the negative *z*-direction onto the metasurface. Here the height H of the PMN-PT rectangular block remained constant at 1.5 μm. Notably, irrespective of the incident light wavelength, the metasurface exhibits the capability to achieve complete 2π phase shift over the incident light by modulating the external voltage U_bias_ while maintaining uniform transmittance. Figure 4c,d illustrate the relationship curves of the transmission coefficient S_21_ amplitude and phase with the external voltage U_bias_ for incident light wavelengths of λ = 1.48 μm and 1.53 μm, respectively. Within these figure panels, the green dots represent data points where the phase difference between adjacent points on the transmission coefficient phase curve amounts to 45°.

Subsequently, a PMN-PT metasurface composed of eight identical structure units was constructed, with external voltages corresponding to the green dots in Figure 4c,d sequentially applied to these eight units. The simulations produce the electric field *E_x_* distribution of the transmitted light wave for the metasurface, as demonstrated in Figure 4e,f, corresponding to incident light wavelengths of λ = 1.48 μm and 1.53 μm, respectively. Specifically, for the metasurface in Figure 4e, these voltages (−475 V, −305 V, −175 V, −50 V, 140 V, 350 V, 570 V, 785 V) were applied sequentially to its structure units; for the metasurface in Figure 4f, these voltages (−720 V, −535 V, −375 V, −260 V, −70 V, 110 V, 325 V, 555 V) were applied sequentially to its structure units. In these two scenarios, the measured deflection angles were 3.62° and 3.80°, closely mirroring the values of 3.65° and 3.77° calculated using the generalized Snell’s law [1]. This simulation strongly underscores the feasibility of freely modulating the operating wavelength of the PMN-PT beam-deflecting metasurface in the optical communication band through simple adjustment of the external voltage. The regulation external voltage of this tunable metasurface is slightly large, but it may be solved by designing a reflective metasurface structure [39]. This will be explored in our follow-up study.

**Figure 5:**
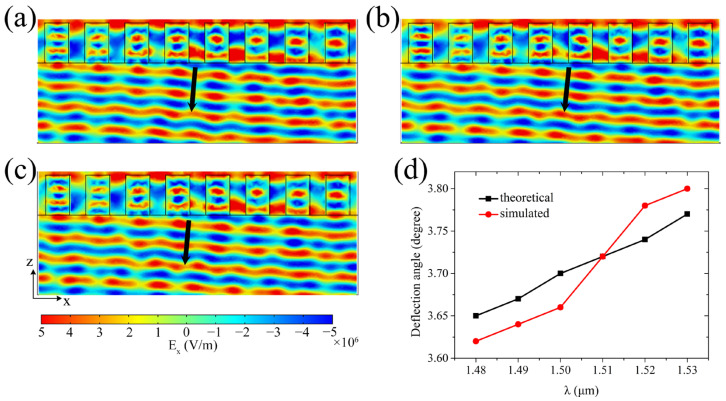
Relationship between the deflection angle and the wavelength of incident light. (**a**–**c**) Electric field *E_x_* distribution of the transmitted light for *x*-polarized light at wavelengths of λ = 1.49 μm, 1.51 μm, and 1.52 μm, respectively. (**d**) Relationship between the deflection angle and the wavelength of incident light. The simulated deflection angles (the red circle dot) were measured from the transmission electric field distributions. The black square dots represent the theoretical deflection angles calculated by using the generalized Snell’s law [1].

Moreover, the data presented in Figure 4a,b imply that beam deflection of the metasurface at other wavelengths can be attained by controlling the external voltages applied in the units. Thus, when the wavelength of incident light is 1.49 μm, 1.51 μm, or 1.52 μm, the transmission electric field distributions of the metasurface with a 45° phase difference between adjacent units are simulated and shown in Figure 5a–c, with the deflection angles measured by using a protractor, as shown by the red circle dots in Figure 5d. Specifically, for the metasurface in Figure 5a, these voltages (−540 V, −365 V, −225 V, −100 V, 80 V, 280 V, 500 V, 720 V) were applied sequentially to its structure units; for the metasurface in Figure 5b, these voltages (−665 V, −490 V, −325 V, −210 V, −25 V, 155 V, 365 V, 590 V) were applied sequentially to its structure units; for the metasurface in Figure 5c, these voltages (−725 V, −555 V, −380 V, −265 V, −80 V, 90 V, 295 V, 520 V) were applied sequentially to its structure units. For comparison, Figure 5d contains the results when the wavelength of incident light was 1.48 μm, 1.5 μm, or 1.53 μm. By comparing the theoretical values (the black square dots in Figure 5d), it is found that the deflection angle measured from the simulation results was nearly consistent with the theoretical prediction, and its value increased with the increase of wavelength. Therefore, these simulations also indicate that the operating wavelength of the beam-deflecting metasurface is freely tunable by simply adjusting the external voltage applied to the PMN-PT rectangular blocks without necessitating any modifications to the structure or geometric parameters of the metasurface.

It can be seen from Figure 1c that the reflectance of the metasurface only has a high value in a very narrow band, and we skipped this band through selecting appropriate external voltage, so that the transmittance of the metasurface was maintained at a high value, as shown by Figure 2c and Figure 4c,d. Therefore, the reflectance is reduced to less than 10% by selecting appropriate external voltages, so as to reduce the influence of reflection on the transmitted light of the metasurface. In this paper, the maximum external voltage applied to the metasurface structure was about 700 V per 1.5 μm, which is slightly larger than the 100 V per 1.5 μm reported in the literature [39], but may be reduced by designing reflective metasurface structure. It is known from the literature [46,47] that PMN-PT crystals with high electro-optic coefficients also have a high piezoelectric coefficient, so the application of external voltage will inevitably lead to structure deformation, which will affect the optical response behavior of the structure. Because deformation of PMN-PT crystal under the influence of piezoelectric effect is complex, these deformations were not considered in the simulations performed in this paper. And since the size L of the structure along the *y*-direction is equal to its period P in simulation, and the boundary conditions in the *y*-direction are periodic boundaries, the size change of the structure along the *y*-direction caused by the piezoelectric effect has little effect on the optical response behavior by applying the external voltage proportionally. The optical response behavior of the metasurface structure is mainly affected by the height H and width W, which was demonstrated by simulation. In the subsequent experiments, the piezoelectric effect may lead to a change in the size of the structure, and then affect the applied voltage value, so in order to achieve the desired performance, we need to slightly adjust the applied voltage by studying the change law of the size. Furthermore, PMN-PT films are required for the preparation of metasurfaces, and PMN-PT films has not been commercialized yet and have only been reported in some laboratories, so we failed to conduct actual metasurface sample preparation and experimental verification. If a suitable PMN-PT film material is found subsequently, we should be able to fabricate the metasurface with only a slight adjustment of the structure. Nevertheless, a beam-deflecting metasurface with an adjustable operating wavelength and deflection angle was proved by simulation using a PMN-PT crystal with a high electro-optic coefficient in this paper, which lays a good theoretical foundation for subsequent experimental research.

## 3. Materials and Methods

All the transmission and reflection spectra, the relationships between the transmission coefficient S_21_ and incident light wavelength λ and external voltage U_bias_, and the electric field *E_x_* distributions of the transmitted light waves for the metasurfaces were simulated via COMSOL Multiphysics 5.5 software. The boundaries of the metasurface structure along the *x*- and *y*-directions were governed by Floquet periodic conditions, while the *z*-direction was equipped with incident and exit ports, incorporating perfect matched layers at the ports. The *x*-polarized light was perpendicularly incident to the metasurface from the negative *z*-direction.

## 4. Conclusions

In summary, a PMN-PT crystal distinguished by a substantial electro-optic coefficient of 120 pm/V was employed to design a simple rectangular metasurface in this paper, and its electrically tunable performance in the optical communication band was proved by simulation. By optimizing the structure parameters and adjusting the external voltage, a beam-deflecting metasurface which can generate a complete 2π phase shift and the relatively uniform transmitted light was designed. Subsequent simulations validated its ability to electrically tune the deflection direction and operating wavelength in the optical communication band. By adjusting the external voltages of the structure units, the deflection angle of the metasurface could be changed from 3.66° to 14.90°, and the operating wavelength could be changed from 1.48 μm to 1.53 μm. Of course, if the medium in the transmission space is changed to air, we will obtain larger deflection angles, and we can also continue to adjust the applied voltages of the structure units to make the metasurface work at other wavelengths. The straightforwardness and ease of fabrication associated with this tunable optical metasurface hold significant importance for broadening the application range of metasurfaces while concurrently contributing to substantial reductions in the manufacturing costs of optical metasurfaces. Consequently, this innovation holds considerable potential for widespread adoption across diverse domains, including optical communication and imaging.

## Data Availability

The original contributions presented in the study are included in the article; further inquiries can be directed to the corresponding authors.
